# Differences in severity at admission for heart failure between rural and urban patients: the value of adding laboratory results to administrative data

**DOI:** 10.1186/s12913-016-1380-z

**Published:** 2016-04-18

**Authors:** Mark W. Smith, Pamela L. Owens, Roxanne M. Andrews, Claudia A. Steiner, Rosanna M. Coffey, Halcyon G. Skinner, Jill Miyamura, Ioana Popescu

**Affiliations:** Truven Health Analytics, 7700 Old Georgetown Rd, Suite 650, Bethesda, MD 20814 USA; Center for Delivery, Organization and Markets, Agency for Healthcare Research and Quality, 5600 Fishers Lane, Room 07W25C, Mail Stop Number 7W25B, Rockville, MD 20857 USA; Truven Health Analytics, 4819 Emperor Blvd, Durham, NC 27703 USA; Hawai’i Health Information Corporation, 733 Bishop St, Suite 1870, Honolulu, HI 96813 USA; Department of Internal Medicine, University of California Los Angeles, 200 UCLA Medical Plaza, Los Angeles, CA 90095 USA; RAND Corporation, Santa Monica, CA USA

**Keywords:** Heart failure, Severity of illness, Clinical laboratory results, Discharge data, Rural hospitals, Urban hospitals

## Abstract

**Background:**

Rural/urban variations in admissions for heart failure may be influenced by severity at hospital presentation and local practice patterns. Laboratory data reflect clinical severity and guide hospital admission decisions and treatment for heart failure, a costly chronic illness and a leading cause of hospitalization among the elderly. Our main objective was to examine the role of laboratory test results in measuring disease severity at the time of admission for inpatients who reside in rural and urban areas.

**Methods:**

We retrospectively analyzed discharge data on 13,998 hospital discharges for heart failure from three states, Hawai’i, Minnesota, and Virginia. Hospital discharge records from 2008 to 2012 were derived from the State Inpatient Databases of the Healthcare Cost and Utilization Project, and were merged with results of laboratory tests performed on the admission day or up to two days before admission. Regression models evaluated the relationship between clinical severity at admission and patient urban/rural residence. Models were estimated with and without use of laboratory data.

**Results:**

Patients residing in rural areas were more likely to have missing laboratory data on admission and less likely to have abnormal or severely abnormal tests. Rural patients were also less likely to be admitted with high levels of severity as measured by the All Patient Refined Diagnosis Related Groups (APR-DRG) severity subclass, derivable from discharge data. Adding laboratory data to discharge data improved model fit. Also, in models without laboratory data, the association between urban compared to rural residence and APR-DRG severity subclass was significant for major and extreme levels of severity (OR 1.22, 95 % CI 1.03–1.43 and 1.55, 95 % CI 1.26–1.92, respectively). After adding laboratory data, this association became non-significant for major severity and was attenuated for extreme severity (OR 1.12, 95 % CI 0.94–1.32 and 1.43, 95 % CI 1.15–1.78, respectively).

**Conclusion:**

Heart failure patients from rural areas are hospitalized at lower severity levels than their urban counterparts. Laboratory test data provide insight on clinical severity and practice patterns beyond what is available in administrative discharge data.

## Background

A substantial number of hospitalizations have been deemed potentially avoidable [1]. They include admissions for ambulatory care sensitive conditions (ACSCs), a set of illnesses for which appropriate, timely ambulatory care may reduce the need for hospitalization [2, 3]. In particular, many studies have noted the higher rate of ACSC hospitalization for prevalent conditions such as heart failure (HF) in rural areas relative to urban areas [3, 4].

While differences in ACSC hospitalization rates by location have been attributed to differences in access to timely ambulatory care, an alternative explanation for differences in rates relates to admission decisions. Physicians may be likely to admit patients from rural areas with lower clinical severity than patients from urban areas. Rural patients could be admitted at lesser severity as a precaution. For example, if the admitting physician believes that the patient is not obtaining sufficient ambulatory care, would not have access to necessary acute care in a timely manner, or would not obtain adequate ambulatory care following a future hospitalization, then a lower severity threshold for admission may be justified.

Judging severity of illness at admission is difficult with traditional data. Chart reviews provide very detailed information but are prohibitively expensive. As a result, multi-site studies of hospital care have long relied on administrative discharge data. Discharge data captures diagnosis and procedures during a stay using *International Classification of Diseases, 9th Revision, Clinical Modification* (ICD-9-CM) diagnosis codes and procedure codes. The ICD-9-CM codes frequently do not capture the level of severity or the change in severity over the course of treatment. Laboratory results provide a window into patient health day-by-day during hospitalization and are available from the moment of admission. Recent technological advances in the field of electronic data management, and the adoption of a uniform set of codes for laboratory data (known as the Logical Observation Identifiers Names and Codes, or LOINC) [5], have enabled the successful integration of key laboratory test values with hospital discharge data. Despite variable implementation of these standard codes, integrated discharge and laboratory databases hold the promise of improved severity measurement for a broader range of populations and outcomes than has been possible with medical record data. Research has shown that enhancing administrative data with clinical laboratory data collected at hospital admission substantially improves the performance of models estimating risk-adjusted hospital mortality [6–8].

Using data collected on hospitalized patients we cannot directly evaluate the role of severity in the decision to admit to the hospital because we lack data on patients who were not admitted. Nevertheless, we can observe variations in the level of severity among newly admitted patients, and whether severity on admission as assessed by laboratory values varies across patients from rural versus urban areas. In the current study, we sought to investigate whether patients from rural areas were admitted to hospitals at lower severity levels than patients from urban areas and whether laboratory values at hospital admission would reflect such a differential.

## Methods

### Data sources and study cohort

Our analysis was based on a convenience sample of administrative hospital discharge data from the State Inpatient Databases (SIDs), a component of the Healthcare Cost and Utilization Project (HCUP) sponsored by the Agency for Healthcare Research and Quality (AHRQ), and on hospital laboratory data assembled for three states during prior AHRQ-supported grants and contracts [9, 10]. We used 2008 Virginia SID data, 2010–2011 Hawai’i SID data, and 2010–2012 Minnesota SID data to identify patients with a principal diagnosis of HF (ICD-9-CM codes 398.91, 402.01, 402.11, 402.91, 404.01, 404.03, 404.11, 404.13, 404.91 or 428.xx) in the subset of hospitals for which we were able to link laboratory data and discharge data. Laboratory and discharge records were matched using deterministic linkage, although the specific data elements used varied by state including hospital identifier, encrypted patient identifier, admission date, gender, date of birth and medical record number. We included all HF discharge records for every hospital that could provide more than 10 cases in a calendar year. We limited the file to discharges that occurred in the same calendar year as admission because labs might not link correctly to partially observed stays. Following linkage to laboratory data there were 14 Virginia hospitals in 2008; in Hawaii, 14 hospitals in 2010 and 15 in 2011; and in Minnesota, 4 in 2010, 22 in 2011, and 23 in 2011.

#### Variables

The analysis file included information on patient demographics, ICD-9-CM diagnosis and procedures, All Patient Refined Diagnosis Related Groups (APR-DRG) Complexity Subclass (3M Health Information Systems, Salt Lake City, UT), and year of admission. The maximum number of diagnoses on the record was 18 in Virginia, 20 in Hawai’i, and from 30 to 51 in Minnesota depending on year.

The primary variable of interest was rural/urban location of the patient’s county of residence. To define rural/urban location, we used the 2003 Urban Influence Codes (UIC), a classification of metropolitan and nonmetropolitan U.S. counties published by the U.S. Department of Agriculture [11]. Urban counties were those defined by UIC as metropolitan areas. Rural counties included micropolitan and all other non-metropolitan counties.

We used ICD-9-CM secondary diagnosis codes and algorithms developed by Elixhauser et al. [12] to measure comorbid conditions. On the basis of previously published HF risk-adjustment models [13], we retained a set of nine comorbidity indicators for our analyses: diabetes (with and without complications), depression and psychotic disorders, liver disease, paralysis, peripheral vascular disease, chronic pulmonary disorders, renal failure, metastatic cancer, and weight loss disorder. Using the APR-DRG Complexity Subclass, which was originally derived from discharge diagnoses recorded on the discharge record, we classified discharge stays by the extent of physiologic decompensation or organ system loss of function: minor loss (1), moderate loss (2), major loss (3), and extreme loss (4) [14]. The diagnosis codes are established at discharge and so may reflect clinical events that occurred throughout the hospital stay.

Other hospital discharge variables included in the analyses were patient age group (18–44, 45–64, 65–84, and 85 or older), gender, and year of admission.

#### Laboratory measures of heart failure severity

We performed a comprehensive review of prior studies evaluating HF prognostic models [15–26] to identify laboratory measures of HF severity. Those studies examined a range of outcomes associated with severity, including short- and long-term mortality and need for heart transplant or ventricular assistive device (VAD) placement in patients with advanced HF. We selected laboratory tests which have been widely and definitively associated with important clinical outcomes. Final analyses included sodium, hemoglobin, renal function measured as serum creatinine, and B-type natriuretic peptide (BNP).

To measure severity at admission, we only included tests performed on the admission day or up to 2 days in advance. If a patient had more than one result for a particular test during the given time period, we selected the most abnormal value.

For each discharge record, we excluded unrealistic laboratory test values deemed to be incompatible with life. Test thresholds were reviewed by our team of clinicians and selected in accordance to clinical experience and current literature. We then excluded records with all invalid or all missing laboratory data, about one percent of records.

For each of the selected laboratory tests we created three severity categories: *normal*, *abnormal*, and *severely abnormal*. The threshold values used for defining these categories were adapted from values used in scores previously developed to predict disease severity in published literature. Where possible, we employed cutoffs similar to those used in the development of the Acute Physiologic and Chronic Health Evaluation (APACHE) II and III score [27]. Because the APACHE score uses hematocrit and our study sample hospitals reported only hemoglobin, we applied a common transformation (hemoglobin = hematocrit / 3) to derive hemoglobin cutoff points. BNP is not in the APACHE score, and so we defined cutoff points based on the observed distribution of test results and literature suggesting a linear relationship between BNP and various outcomes [28]. We chose to model missingness directly because there is no reason to assume that the tests, had they been ordered, would have fallen in the normal or abnormal ranges. Table 1 presents the range associated with each category for selected laboratory tests.

**Table 1:** Normal, abnormal, and severely abnormal ranges for selected laboratory tests

Blood elements	Unit	Normal range	Abnormal range	Severely abnormal range
Hemoglobin (female)	g/dl	12–15	7–11	<7
Hemoglobin (male)	g/dl	13–17	7–12	<7
Sodium	mmol/l	137–145	121–136 or 146–160	<121 or >160
Serum creatinine	mg/dl	<1.5	1.5–7.3	>7.3
B-type natriuretic peptide (BNP)	ng/ml	<430	430–1729	>1729

#### Statistical analyses

We examined bivariate descriptive statistics of age, gender, comorbidities, APR-DRG severity subclass, and laboratory test values by patient location. Next we used logistic regression to investigate associations between patient characteristics and the urban/rural classification. Using rural residence as the reference category, we found the relative odds of patients living in urban areas presenting with particular clinical and sociodemographic characteristics at hospital admission. Of particular interest was the association between rural or urban residence and severity of illness at admission, as evidenced by laboratory values.

We estimated the models twice, once with laboratory data and once without. The value of adding laboratory data was judged by three factors: the statistical significance of individual variables; the change in goodness of fit overall for the equation, as measured by Akaike’s Information Criterion (AIC); and whether there were notable changes in the level or significance of other variables caused by adding the laboratory tests [29]. All analyses were performed using SAS statistical software.

The study was performed by (authors PO, RA, CS) and on behalf of AHRQ. The Agency does not require Institutional Review Board approval of analyses of HCUP SID files.

## Results

Across the three states, a total of 13,998 HF discharges met our inclusion criteria and could be matched to at least one valid laboratory test. They represented roughly 85 % of HF discharges in the selected hospitals.

The characteristics of patients included in the study sample are summarized in Table 2, by urban/rural category. A small number of invalid values was set to missing. Most patients resided in metropolitan areas (75.7 %; *N* = 10,593). Relative to patients from rural areas, those from urban areas were younger on average (*p* < .0001) and more likely to be female (48.9 % vs. 43.8 %, *p* < .0001). People from urban areas were more likely to be diagnosed with hypertension (67.5 % vs. 64.8 %, *p* < .05), metastatic cancer (1.1 % vs. 0.7 %, *p* < .05), and weight loss disorders (3.6 % vs. 1.5 %, *p* < .0001) but less likely to be diagnosed with diabetes without complications (34 % vs. 32 %, *p* < .05) and chronic pulmonary disease (30.1 % vs. 33.2 %, *p* < .001).

**Table 2:** Descriptive statistics by rural/urban residence of patient

	Urban		Rural		p-value
	N	%	N	%	
Number of discharges	10,593	75.7	3,405	24.3	
Patient characteristics					
Age group (years)					<.0001
18–44	547	5.2	130	3.8	
45–64	2,504	23.6	676	19.9	
65–84	4,889	46.2	1,746	51.3	
85+	2,653	25.0	853	25.1	
Gender					<.0001
Female	5.185	48.9	1,491	43.8	
Male	5,408	51.1	1,914	56.2	
AHRQ Comorbidity Indictors					
Diabetes without chronic complications	3,350	31.6	1,140	33.5	.0436
Diabetes with chronic complications	1,161	11.0	372	10.9	.9547
Hypertension	7,152	67.5	2,208	64.8	.0040
Depression	843	8.0	288	8.5	.3516
Pulmonary circulation disorders	29	0.3	--^a^	--	--
Peripheral vascular disease	1,044	9.9	318	9.3	.3765
Paralysis	186	1.8	61	1.8	.8908
Chronic pulmonary disease	3,193	30.1	1,131	33.2	.0007
Renal failure	4,276	40.4	1,400	41.1	.4383
Liver disease	212	2.0	61	1.8	.4411
Metastatic cancer	116	1.1	23	0.7	.0317
Weight loss	377	3.6	50	1.5	<.0001
APR-DRG Severity					<.0001
1 Minor	726	6.9	261	7.7	
2 Moderate	4,106	38.8	1,462	42.9	
3 Major	4,770	45.0	1,446	42.5	
4 Extreme	991	9.4	236	6.9	
Laboratory data					
Hemoglobin					<.0001
Normal	7,594	71.7	2,362	69.4	
Abnormal	2,405	22.7	787	23.1	
Severely abnormal	95	0.9	24	0.7	
Missing	499	4.7	232	6.8	
BNP					
Mean	8,457	1,037	2,294	939	<.0001
25th percentile	8,457	527	2,294	488	
75th percentile	8,457	2,037	2,294	1,782	
Normal	2,018	19.1	596	17.5	<.0001
Abnormal	4,054	38.3	1,163	34.2	
Severely abnormal	2,129	20.1	483	14.2	
Missing	2,392	22.6	1,163	34.2	
Sodium					<.0001
Normal	9,694	91.5	3,081	90.5	
Abnormal	597	5.6	175	5.1	
Severely abnormal	75	0.7	28	0.8	
Missing	227	2.1	121	3.6	
Serum creatinine					.0001
Normal	5,874	55.5	1,819	53.4	
Abnormal	2,188	20.7	780	22.9	
Severely abnormal	2,247	21.2	678	19.9	
Missing	284	2.7	128	3.8	

*BNP* B-type natriuretic peptide

^a^Cell sizes less than 10 are suppressed by HCUP guidelines

Patients from rural areas had lower severity of illness as measured by the APR-DRG Severity Subclass, indicated by higher likelihoods of being in classes 1 (minor) and 2 (moderate) (*p* < .0001).

Differences in the distribution of laboratory results were highly significant for all four selected laboratory tests: hemoglobin, BNP, sodium, and serum creatinine. Patients from rural areas had a higher proportion of missing laboratory data than did those from urban areas, particularly for BNP (34.2 % vs. 22.6 %, *p* < .0001). People in urban areas had higher mean BNP values (1,037 vs. 939, *p* < .0001) and consequently were more likely to have abnormal and severely abnormal BNP values. They were also slightly more likely to have abnormal or severely abnormal sodium values and more likely to have a severely abnormal creatinine value (21.2 % vs. 19.9 %, *p* = .0001).

Table 3 shows the regression adjusted odds ratios. Results in the first two columns on the left derive from models with demographic and administrative claims data; results on the right two columns come from a model with those variables plus laboratory data. The odds ratios represent the relative odds of being from residing in an urban area relative to a rural area. Values above 1.0 indicate greater odds of living in an urban area.

**Table 3:** Logistic regression of living in urban versus rural areas

Effect	Without laboratory data			With laboratory data		
	Odds Ratio	95 % Wald		Odds Ratio	95 % Wald	
		Confidence Limits			Confidence Limits	
Patient demographics						
Age (years)						
18–44	reference			reference		
45–64	.933	.774	1.13	.911	.754	1.10
65–84	.652	.548	.777**	.645	.540	.770**
85+	.736	.612	.886**	.714	.592	.863**
Female	1.22	1.12	1.32**	1.20	1.11	1.31**
AHRQ comorbidity indicators						
Hypertension	1.26	1.16	1.38**	1.23	1.12	1.34**
Renal failure	.986	.903	1.08	1.05	.940	1.17
Chronic lung disease	.829	.761	.904	.821	.752	.897**
Diabetes mellitus	.922	.848	1.00	.932	.856	1.01
Weight loss	2.48	1.83	3.36**	2.50	1.84	3.39**
Paralysis and other neurological disorders	.874	.753	1.00	.887	.763	1.03
Metastatic cancer	1.51	.953	2.38	1.46	.915	2.31
Peripheral vascular disease	1.11	.972	1.28	1.11	.964	1.27
Depression and psychoses	1.08	.946	1.23	1.06	.931	1.21
Liver disease	1.11	.830	1.50	1.10	.819	1.49
Characteristics of the stay						
Year						
2008	2.95	2.58	3.37**	3.60	3.31	4.14**
2010	1.47	1.29	1.67**	1.36	1.20	1.55**
2011	1.12	.992	1.26	1.07	.942	1.21
2012	reference			reference		
APR-DRG Severity						
1	reference			reference		
2	1.01	.861	1.19	.918	.779	1.08
3	1.22	1.03	1.43*	1.12	.942	1.32
4	1.55	1.26	1.92**	1.43	1.15	1.78**
Laboratory results						
Hemoglobin – abnormal				.957	.869	1.06
Hemoglobin – severely abnormal				1.07	.673	1.71
Hemoglobin – missing				.922	.765	1.11
Sodium – abnormal				1.10	.922	1.32
Sodium – severely abnormal				.831	.527	1.31
Sodium – missing				.799	.487	1.31
Creatinine – abnormal				.811	.723	.909**
Creatinine – severely abnormal				.917	.801	1.05
Creatinine – missing				1.17	.730	1.86
BNP – abnormal				1.05	.931	1.17
BNP – severely abnormal				1.29	1.12	1.48**
BNP – missing				.461	.407	.522**
AIC	14,981.9			14,643.1		

* *p* < .05. ** *p* < .01

*BNP* B-type natriuretic peptide, *AIC* Akaike’s Information Criterion

Odds ratios and the pattern of significance were similar across the models for demographic and comorbidity variables. Among patients admitted with HF in this sample, people from urban areas were less likely 65 and above, more likely female, and more likely to present with APR-DRG major and extreme severity subclass. They were also more likely to have hypertension and weight loss, and less likely to have chronic lung disease.

Using APR-DRG Severity level 1—lowest severity—as the reference, patients from urban areas were more likely to be in categories 3 (OR 1.22, *p* < .05) and 4 (OR 1.55, *p* < 01) in the model without laboratory results. When laboratory data were added, only the highest category remained statistically significant, although its effect was attenuated (OR 1.43, *p* < .01).

Three of the 12 laboratory values were statistically significant but without a clear pattern. Individuals from urban areas were less likely to have abnormal creatinine values (OR .811, *p* < .01) or missing BNP test (OR .461, *p* < .01). Among patients with valid BPN values, however, urban patients were more likely to have severely abnormal BNP values (OR 1.29, *p* < .01). There were no significant differences in the proportions of normal and abnormal hemoglobin or sodium levels, or on levels of missingness of these two tests.

Adding laboratory variables meaningfully improved model fit, as reflected by a decrease in the AIC of 338 points. (A decrease of 9.2 points or greater indicates that the model without laboratory values has a chance of less than 1% of being the better model.) There was little impact on the level or significance of the odds ratios for the other independent variables.

## Discussion

Differences in HF severity at admission across rural and urban areas could have implications for the evaluation of programs to reduce avoidable hospitalizations, and so possible underlying explanations are important to consider. Although our analysis found that, on average, rural patients have lower severity on admission, the severity range could still be similar for rural and urban patients; rural patients with less severity may be admitted more often. For important reasons, including long travel distances and limited community resources, hospitals may admit residents of rural areas at a lower severity threshold than for residents of urban areas. In practice it is often difficult for doctors to discharge patients from the emergency department who have traveled for an hour or more from a remote rural area to reach the hospital, very likely at the advice of a community doctor or nurse practitioner. Moreover, there is always a degree of uncertainty in medical decisions over how severe the HF decompensation is and whether it can be managed at home. In this scenario, improving ambulatory care could have a significant impact on preventable hospitalization rates. Better management in the outpatient setting (e.g., through outpatient multidisciplinary HF programs and telemedicine) would reduce HF exacerbations that require hospitalization.

An alternative explanation is that rural hospitals admit fewer terminally ill patients. This could arise, for example, if rural patients have lower rates of hospital utilization for end-stage disease. Analyses performed by the Dartmouth Atlas research team have shown large regional variations in the utilization of hospital and intensive care units for end-of-life-care, across states and metro area hospital markets and sometimes even for hospitals within the same market, although whether these differences are due to differences in utilization between rural and urban populations is unknown [30]. Nevertheless, it is unlikely that home health and hospice resources in remote rural areas are adequate to handle severe cases outside the hospital setting. Increasing outpatient resources (e.g., nursing and case management staff) and education and training efforts in rural areas could create more effective primary care networks, able to manage HF patients successfully in the ambulatory setting and prevent exacerbations. Effective ambulatory settings should also be equipped to treat and reduce hospitalizations for lower-severity HF exacerbations, more common in rural settings as our study suggests.

Laboratory data could be incorporated in hospital initiatives to improve quality for HF treatment and reduce rates of preventable hospitalizations. As our study shows, adding laboratory data to hospitalization records explained some of the differences in severity by APR-DRG Severity class, suggesting that laboratory data are indeed correlated with and could be used as a measure of severity that, represents a snapshot of patients’ health at the time of hospital presentation. It is likely that more detailed clinical information beyond laboratory data may enhance the ability of a model predicting severity, but such data would be very difficult and expensive to collect and centralize routinely at this time.

Finally, people from rural areas were less likely than others to receive certain laboratory tests, such as hemoglobin or BNP, before or at admission. A link between rural/urban residence and the likelihood of being given a test could arise from several causes. For example, rurality of residence most likely correlates positively with rurality of hospital, and rural and urban hospitals may differentially use laboratory tests. Rural hospitals may be more likely to transfer high-acuity patients quickly, before many laboratory tests can be ordered. Lastly, ordering more expensive tests (such as BNP) might not have been deemed necessary to successfully manage patients with relatively low severity and good short-term prognosis. Regardless of the cause, this level of missingness for some laboratory tests likely represents differences in provider practice patterns.

We acknowledge several limitations. First, the relation of rural/urban location to patient characteristics could be considerably different for conditions other than HF. Second, the absence of outpatient encounter and pharmacy data linked to these inpatient records reduced our ability to judge severity of illness at admission. Third, our data comprised records from a convenience sample of hospitals in three states. Although most Hawai’i hospitals participated, the proportions were relatively low in Virginia and Minnesota. Results could vary in a different set of states or with a fuller representation of hospitals. Our models could not fully control for differences between urban and rural residents; a richer set of control variables could affect the relation of lab results to our outcomes. Finally, in our sample 75.7 % of HF stays were for individuals from metropolitan areas. We do not have similar data on the urbanicity of HF patients in other states, but we can compare this figure to the overall urbanicity of each state. The Census Bureau updated the Urban Influence Codes in 2010, and so 2000 was the only decennial census year to which the 2003 UICs were applied. In 2000, 72.3 % of Hawai’i residents, 70.4 % of Minnesotans, and 78.1 % of Virginians lived in metropolitan areas as we define them. These percentages bracket the average in our sample of people with HF discharges.

## Conclusion

Using combined administrative and laboratory data, we found that, among HF patients, rural residence was associated with lower APR-DRG Severity Class and lower odds of having a severely abnormal admission BNP, the most HF-specific laboratory severity marker. These findings point to lower severity at hospital admission among HF patients from rural areas relative to those from urban areas. The finding is important in that it suggests that improving ambulatory resources to care for HF or improving patient education in rural areas may indeed reduce unnecessary hospitalizations. Moreover, we found that laboratory data does indeed reflect differences in severity at admission. Given the increasing availability of automated laboratory data, this important resource can be tapped in local quality improvement efforts by hospitals and clinicians.

## Availability of data and materials

HCUP State Inpatient Databases are publicly available for purchase. See http://www.hcup-us.ahrq.gov/sidoverview.jsp for details. Laboratory results data used in these analyses have restricted access and require special permission. Requests for access should be directed to AHRQ at hcup@ahrq.gov.

## Abbreviations

ACSC: Ambulatory care sensitive condition
AIC: Akaike’s Information Criterion
APACHE: Acute Physiologic and Chronic Health Evaluation
APR-DRG: All-Payer Refined Diagnosis-Related Group
AHRQ: Agency for Healthcare Research and Quality
BNP: B-type natriuretic peptide
HF: Heart failure
LOINC: Logical Observation Identifiers Names and Codes
HCUP: Healthcare Cost and Utilization Project
SID: State Inpatient Databases
VAD: Ventricular assistive device
